# Case Report: BCG-Triggered Hemophagocytic Lymphohistiocytosis in an Infant With X-Linked Recessive Mendelian Susceptibility to Mycobacterial Disease Due to a Variant of Chronic Granulomatous Disease

**DOI:** 10.3389/fped.2021.687538

**Published:** 2021-06-29

**Authors:** Suleiman Al-Hammadi, Amal M. Yahya, Abdulla Al-Amri, Amar Shibli, Ghazala B. Balhaj, Mohamed I. Tawil, Ranjit Vijayan, Abdul-Kader Souid

**Affiliations:** ^1^College of Medicine, Mohamed Bin Rashid University of Medicine and Health Sciences, Dubai, United Arab Emirates; ^2^Department of Pediatrics, College of Medicine and Health Sciences, United Arab Emirates University, Al Ain, Abu Dhabi, United Arab Emirates; ^3^Department of Pediatrics, Tawam Hospital, Al Ain, Abu Dhabi, United Arab Emirates; ^4^Department of Pediatrics, Al Ain Hospital, Al Ain, Abu Dhabi, United Arab Emirates; ^5^Department of Radiology, Sheikh Khalifa Medical City, Abu Dhabi, United Arab Emirates; ^6^Department of Biology, College of Science, United Arab Emirates University, Al Ain, Abu Dhabi, United Arab Emirates

**Keywords:** Bacillus Calmette-Guérin, BCG disease, vaccines, tuberculosis, immunodeficiency, inborn error of immunity, newborns, United Arab Emirates

## Abstract

In the United Arab Emirates, BCG (Bacillus Calmette-Guérin) is administered to all newborns. We present here a young infant with an inborn error of immunity (IEI) who developed fatal adverse events to this live-attenuated vaccine. This male infant received BCG (Serum Institute of India Pvt., Ltd., India) on Day 11 of life. On Day 25, he developed fever, followed by cervical lymphadenitis and bilateral otitis media with fluid drainage. On Day 118, he was admitted with severe hemophagocytic lymphohistiocytosis (HLH), and passed away on Day 145. The diagnostic exome sequencing test identified a hemizygous nonsense variant, NM_000397.3(*CYBB*):c.676C>T, p.Arg226^*^ (rs137854592). Pathogenic variants of *CYBB* [cytochrome b(-245), beta subunit; Mendelian Inheritance in Man [MIM] accession code, 300481] are known to cause “immunodeficiency 34, mycobacteriosis, X-linked” (IMD34, MIM#300645) and “chronic granulomatous disease, X-linked” (CGDX, MIM#306400). The natural history of his illness is consistent with “*X-linked recessive Mendelian susceptibility to mycobacterial disease (MSMD)*.” This entity is responsible for his BCG disease and is a likely trigger of his HLH. This disastrous event underlines the importance of developing worldwide policies that target BCG disease prevention, especially in communities with high prevalence of IEI. Moreover, screening for genetic causes of MSMD in the community could pave the way, at least partially, for scale-up of tuberculosis (TB) prevention.

## Introduction

Bacillus Calmette-Guérin, a live vaccine derived from attenuated *Mycobacterium bovis* cultures, is administered to all newborns in the United Arab Emirates (UAE) since 2005 (1). This practice has resulted in serious adverse events in our tribal communities, especially in children with unrevealed inborn error of immunity (IEI) (2, 3). Moreover, the BCG strains may also cause serious (often fatal) complications in “*Mendelian susceptibility to mycobacterial diseases*” (MSMD) (4). MSMD embraces inherited entities that result from both “virulent” and “faintly virulent” mycobacteria, including BCG vaccines (5, 6). An example of an MSMD-causing autosomal variant in our tribal communities is *IFNGR2* (interferon-gamma receptor 2; MIM#147569):c.123C>G, p.Tyr41^*^, which results in “immunodeficiency 28, mycobacteriosis” (IMD28; MIM#614889) (7). Examples of MSMD-causing X-linked pathogenic variants include *CYBB*:Gln231Pro (rs151344498), *CYBB*:Thr178Pro (rs151344497), and NM_000397.3(*CYBB*):c.1662dup, p.Glu555^*^ (rs1453468510) (8–11). This case report describes serious adverse events of the BCG vaccine in a young infant with X-linked MSMD. Its main aim is to provide further justifications for establishing safe rules for BCG vaccination.

## Case Description

**Figure 1:**
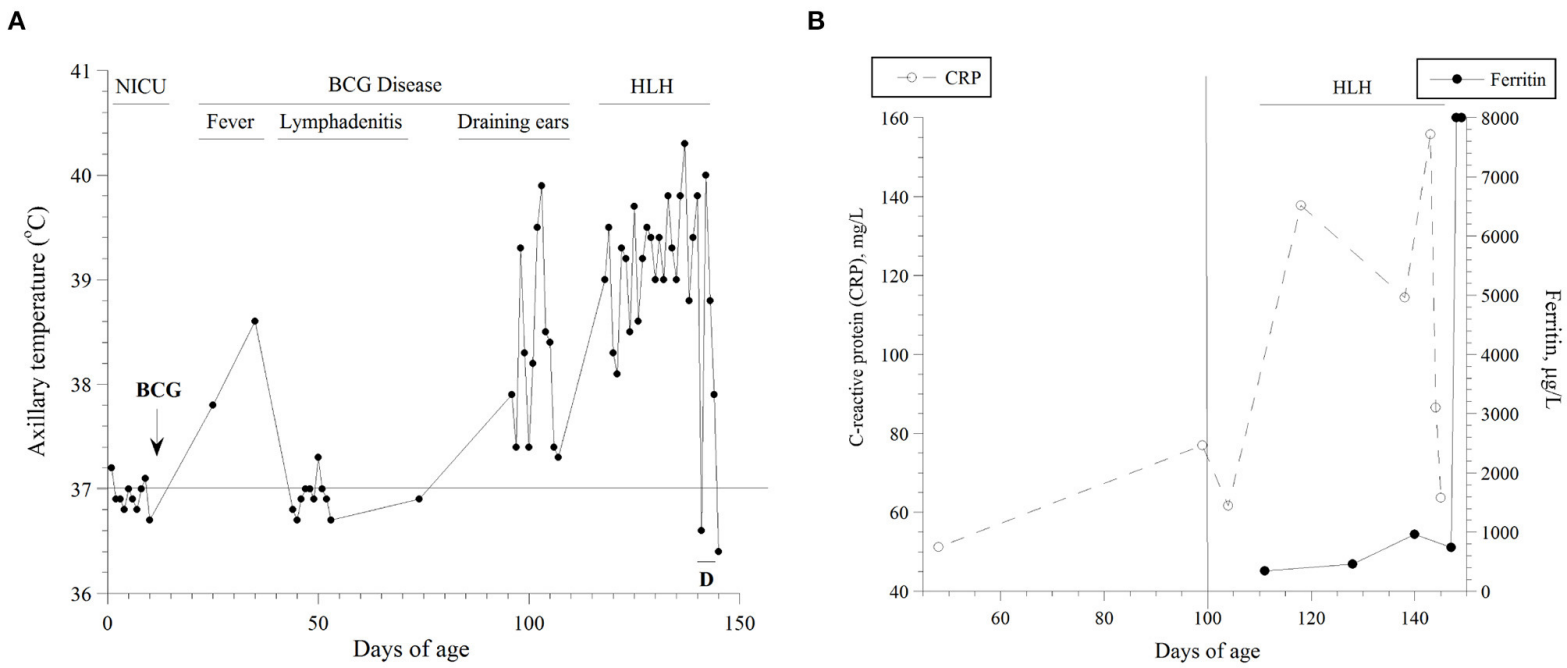
**(A)** Axillary temperatures as function of age. The highest value per day is shown. The natural course of his disease is also depicted. D, dexamethasone; NICU, neonatal intensive care unit; HLH, hemophagocytic lymphohistiocytosis. He was inpatient during the highlighted periods. **(B)** Serum levels of C-reactive protein (CRP, mg/l) and ferritin (μg/l) as functions of age. Estimations of the onset (vertical line on Day 100) and duration of his HLH are depicted.

This male infant, an offspring of first-cousin parents, was born at 35 weeks' gestation by Cesarean delivery due to premature contractions. The mother was gravida 4, para 3. Her pregnancy was complicated by gestational diabetes (managed with insulin) and systemic lupus erythematosus (SLE, treated with hydroxychloroquine). She was on prophylactic enoxaparin during the pregnancy. Otherwise, the family health history (parents, siblings, grandparents, uncles, and aunts, and first-degree cousins) was unremarkable, including negative exposure to known tuberculosis (TB) cases. His birth weight was 3,125 g. His postnatal period was complicated by respiratory distress that required neonatal intensive care supports for 11 days. BCG (0.05 ml, intradermal injection in the upper right arm [manufactured by Serum Institute of India Pvt. Ltd., India]) and hepatitis B pediatric [10 μg [0.5 ml] intramuscular injection] vaccines were then administered on Day 11 (the day of discharge from the neonatal intensive care unit, NICU). Thereafter, he developed unremitting fever (Figure 1A) and passed away on Day 145 (Table 1).

**Table 1:** Diagnostic-treatment timeline for his inpatient periods; values are mean ± SD.

	**Fever-cervical lymphadenitis (Days 25–75)**	**Draining ears (Days 84–108)**	**HLH (Days 118–144)**
Hemoglobin, g/l	88 ± 16	78 ± 11	71 ± 6
Platelet count, 10^9^/l	502 ± 115	363 ± 88	79 ± 64
Neutrophil count, 10^9^/l	10.8 ± 3.9	7.1 ± 2.0	2.4 ± 0.5
Lymphocyte count, 10^9^/l	10.8 ± 3.4	10.0 ± 3.3	1.2 ± 0.7
C-reactive protein, mg/l	37 ± 40	92 ± 40	105 ± 40
Erythrocyte sedimentation rat, mm/h	-	-	100; 102; 59; 2
Ferritin, μg/l	-	399 ± 78	962;739;>8,000;>8,000
Triglyceride, mmol/l	-	-	1.77 ± 0.42
Alanine transaminase, U/l	-	36 ± 6	504 ± 582
Soluble interleukin-2 receptor, pg/ml	-	-	5,386
Hepatosplenomegaly	-	-	Days 118–145
Cytomegalovirus, Epstein–Barr virus, HIV	-	-	Days 121–140
Bone marrow biopsy and spinal tap	-	-	Day 133
Neck ultrasound	Days 47 to 51	-	-
Computerized tomography chest and abdomen	-	-	Day 134
*Staphylococcus haemolyticus*	-	-	Day 141
Intravenous dexamethasone	-	-	Days 140–145
Multiorgan failure/disseminated intravascular coagulation	-	-	144–145

**Figure 2:**
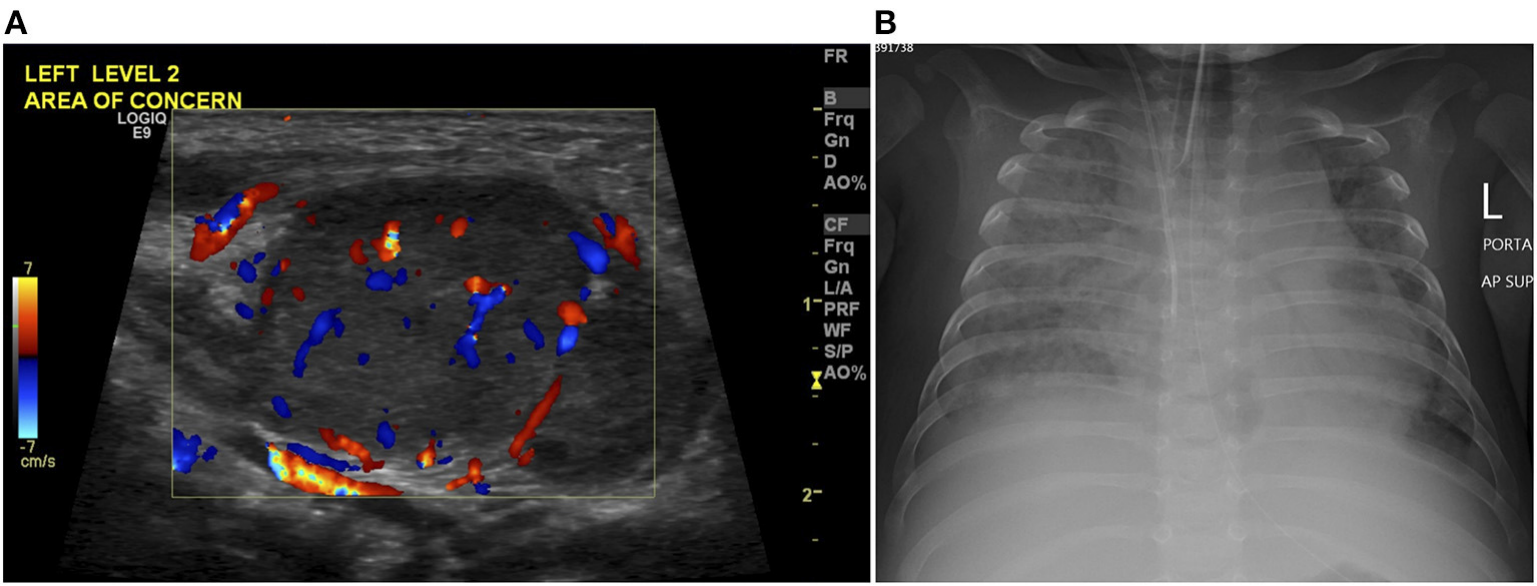
**(A)** Color Doppler images on neck ultrasound scan at age of 45 days demonstrates an enlarged left cervical lymph node of increased vascularity in keeping with lymphadenitis. **(B)** Chest radiograph at age of 145 days shows tube and line in position, and bilateral diffuse alveolar-interstitial opacification with right pleural effusion in keeping with pulmonary edema.

**Figure 3:**
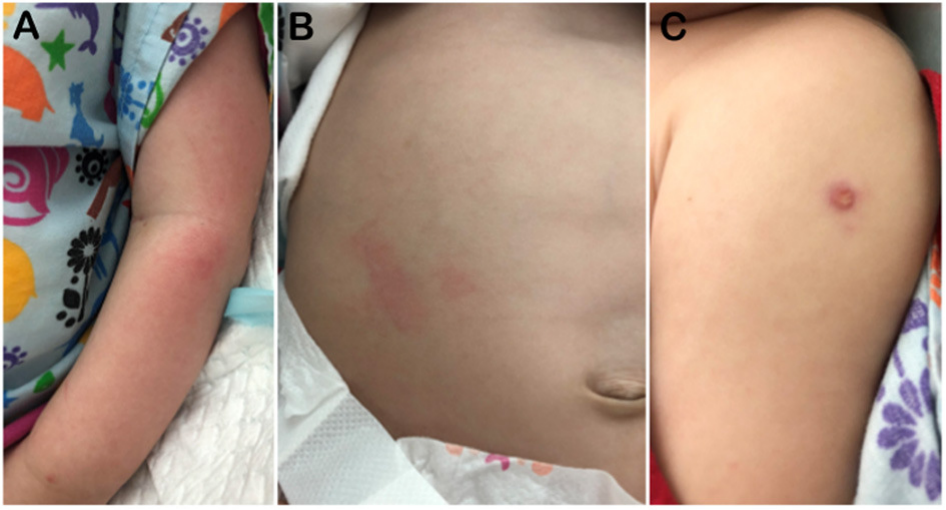
Photographs on Day 140 showing **(A)** his erythematous skin rash, **(B)** massive hepatomegaly, and **(C)** BCG site. The BCG vaccination site measured about 5 mm; it was a wet papule with central yellow crust.

Briefly, he was admitted on Day 45 with fever and bilateral cervical lymphadenitis (a tender left upper cervical lymph node measuring “2.3 × 1.7 × 1.5 cm” and a right cervical lymph node measuring “1.2 × 0.8 × 0.8 cm”; Figure 2A) and received empiric antibiotics for 10 days. On Day 96, he was readmitted for treatment of bilateral otitis media with yellow discharges and received again empiric antibiotics for 10 days. On Day 118, he was admitted with persistent high fever, massive/firm hepatosplenomegaly (spleen 10 cm below the left costal margin and liver down to the pelvis), skin rash, failure to gain weight, and an unhealed BCG injection site (Figure 3). Treatment for BCG disease was never received. On Day 140, he was started on dexamethasone (10 mg/m^2^/days, intravenously), as an empiric treatment of hemophagocytic lymphohistiocytosis (HLH). He passed away on Day 145 of multiorgan (hepatic, respiratory, and renal) failure and disseminated intravascular coagulation (DIC), Figure 2B.

During the last admission, his complete blood counts (CBC), blood film, lymphocyte subset, quantitative immunoglobulin, bone marrow aspirate and biopsy studies (morphology, cytogenetics, and flow cytometry; the results did not reveal active hemophagocytosis), skeletal scintigraphy, and echocardiography were unrevealing (Table 1). Epstein–Barr virus (EBV) and cytomegalovirus (CMV) real-time polymerase chain reaction (PCR) tests were negative. Neutrophil oxygen burst study was not done. Nasopharyngeal swabs for respiratory pathogens were negative by PCR, including coronavirus disease (COVID-19) and severe acute respiratory syndrome coronavirus 2 (SARS-CoV-2).

Initial neck ultrasound scan confirmed presence of a cervical lymphadenitis with no evidence of fluid collection. Computerized tomography (CT) of the chest, abdomen, and pelvis demonstrated hepatosplenomegaly, otherwise were unremarkable. Moderate ascites was demonstrated on follow-up ultrasound scan. Radiographically, he developed a progressive picture of acute respiratory syndrome.

Results of his serum ferritin and C-reactive protein (CRP) are summarized in Figure 1B. Between Days 120 and 145, his serum triglyceride was (mean ± SD) 1.77 ± 0.42 mmol/l (*n* = 5; normal 0.25–0.85), alanine transaminase (ALT) 202 unit/l (increased to 1,175 unit/l on Day 145; normal, ≤ 41), and ammonia 130 μmol/l (normal, 16–60). On Day 130, soluble interleukin-2 receptor (sIL-2R) was 5,386 pg/ml (reference values, 175–858) and interleukin six was 50.6 pg/ml (normal, ≤ 7.0). On Day 143, a blood culture grew *Staphylococcus haemolyticus*. On Day 144, he developed refractory coagulopathy, including hypofibrinogenemia. On Day 147 (two days after he passed away), diagnostic exome sequencing revealed the pathogenic hemizygous variant NM_000397.3(*CYBB*):c.676C>T, p.Arg226^*^ (rs137854592).

## Discussion

This young infant has the hemizygous pathogenic variant *CYBB*:p.Arg226^*^ (rs137854592) and has received the BCG vaccine. Shortly thereafter, he developed BCG disease (fever with lymphadenitis) and progressed to severe HLH (systemic inflammation with massive hepatosplenomegaly due to lymphocyte proliferation). Thus, his HLH is likely triggered by the BCG disease. His treatment must be urgent and directed toward both the BCG disease and HLH. Delaying effective therapies (anti-BCG disease as well as standard HLH management) until a molecular diagnosis is evident is imprudent. It is well to know that the minimum inhibitory concentrations of isoniazid, streptomycin, rifampicin, and ethambutol are included in the BCG vaccine Package Insert (Serum Institute of India Pvt. Ltd., Pune, India).

The term “*Mendelian susceptibility to mycobacterial disease (MSMD)*” labels inherited entities that result from both “virulent” and “faintly virulent” mycobacteria, including BCG vaccines (12–14). *CYBB* (MIM#300481; X-linked) encodes for the beta-subunit of phagocyte NADPH (nicotinamide adenine dinucleotide phosphate) oxidase. Pathogenic variants of *CYBB* such as Gln231Pro (rs151344498) and Thr178Pro (rs151344497) have been identified within this clinical cluster (8). Hemizygous males with these variants have reduced macrophage oxidative burst (a variant of chronic granulomatous disease, CGD), but normal respiratory function in the granulocytes and monocytes. They become symptomatic after exposure to mycobacteria, and their disease has been labeled: “*X-linked recessive MSMD*” (8).

**Figure 4:**
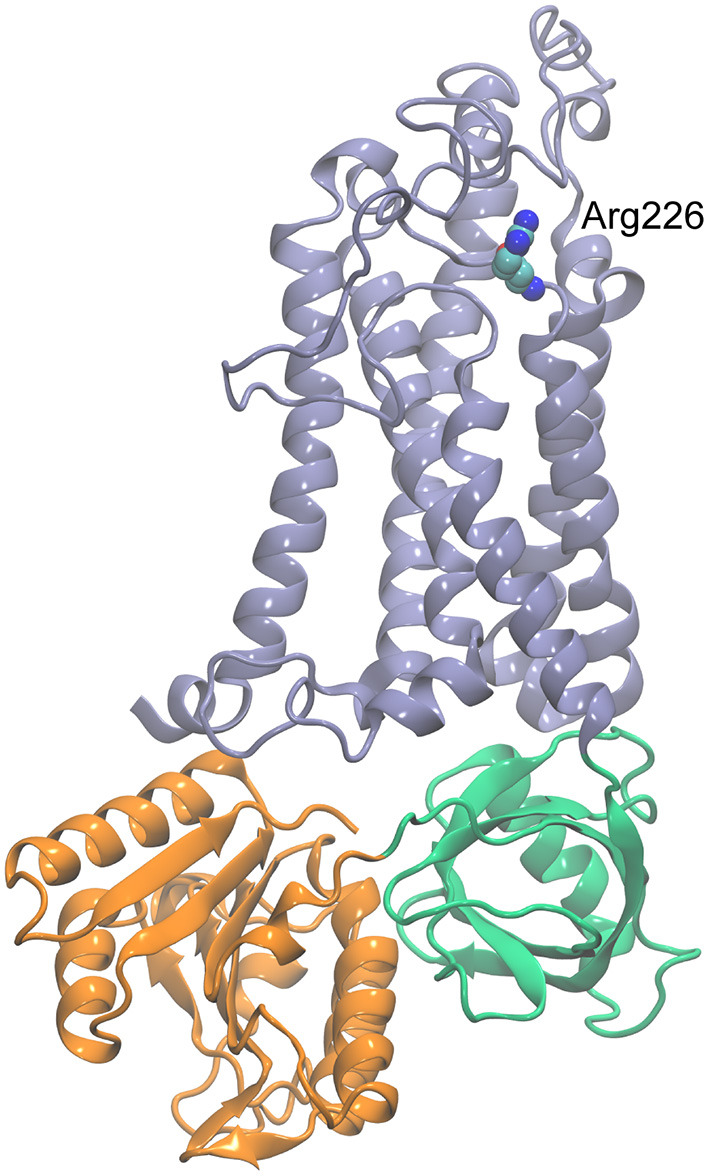
Homology model of CYBB generated with SWISS-MODEL (https://swissmodel.expasy.org/) using the dual oxidase 1 (DUOX1; Protein Data Bank ID: 7D3E) structure as the template. The transmembrane region is shown in blue, FAD-binding domain in green, and NADPH-binding domain in orange. Arg226 is shown in space-filling representation.

Pathogenic variants of *CYBB*, including *CYBB*:p.Arg226^*^ and NM_000397.3(*CYBB*):c.1662dup, p.Glu555^*^ (rs1453468510) (15), are associated with “immunodeficiency 34, mycobacteriosis, X-linked” (IMD34; MIM#300645) and “chronic granulomatous disease, X-linked” (CGDX; MIM#306400) (10, 11). Arg226 is located near the C-terminus end of the fifth transmembrane helix of the protein, and a truncation (non-sense mutation) will delete the cytoplasmic dehydrogenase (DH) domain that consists of the FAD (flavin adenine dinucleotide) and NADPH-binding domains (Figure 4). This deletion will also remove the last transmembrane helix of the protein, likely making it unable to fold or function as expected.

It is also important to point out that the T cell function in these *CYBB* variants is expected to be unaltered. Therefore, a widespread TB disease is unlikely. The problem, rather, is an uncontrolled systemic inflammation, as seen in HLH (16).

Studies are also needed to understand the contribution of IEI variants (i.e., MSMD) to TB disease in our community. Identifying and screening for such variants allow a safer BCG vaccination program and improve the TB screening in individuals at risk (17). Intense migration to UAE from TB endemic regions imposes high risk to the population, especially those with MSMD. In our population, the prevalence of latent TB infection (LTBI) in children is 0.45% and in adults 18.8% (8% among our medical students) (18–20). In addition, future studies are needed to determine whether the carrier state of *CYBB*:p.Arg226^*^ could have contributed to the development of SLE in his mother.

This case report provides further justification for developing and implementing safe rules for BCG vaccination locally and internationally. It is the responsibility of health authorities to establish effective strategies that prevent improper vaccinations. This young infant emphasizes the need to take essential precautions before immunizing children and adults with live vaccines.

It is vital to emphasize the WHO position: “BCG vaccination is contraindicated for persons with congenital cell-mediated or severe combined immunodeficiency” (21). This statement, however, requires further clarification for risks associated with MSMD. Many of the severe forms of IEI manifest clinically in the first few months of life (22). Therefore, in some countries, the BCG vaccine is given to infants at a later age (23–25). Thus, we urge the authorities to refine their statement on BCG and include procedures that assure only healthy children receive the vaccine (26). A summary of the MSMD variants found in the UAE is shown in Table 2.

**Table 2:** Variants of Mendelian susceptibility to mycobacterial disease (MSMD) found in the UAE.

➢ NM_000397.3(*CYBB*):c.676C>T, p.Arg226* (rs137854592) [this report]. ➢ NM_000206.2(*IL2RG*):c.820_823dup p.Ser275Asnfs*29 (novel) causes X-linked severe combined immunodeficiency (MIM#300400) [unpublished]. ➢ NM_178443.2(*FERMT3*):c.1800G>A, p.Trp600* (novel) causes “leukocyte adhesion deficiency, type III” (LAD3, MIM#612840) [unpublished]. ➢ NM_178443.2(*FERMT3*):c.2001del p.*668Glufs*106 (novel) causes “leukocyte adhesion deficiency, type III” (LAD3, MIM#612840) [unpublished]. ➢ *IFNGR2*:c.123C>G, p.Tyr41*; causes “immunodeficiency 28, mycobacteriosis” (IMD28; MIM#614889) [unpublished]. ➢ NM_001287344.1(*BTK*):c.80G>A, p.Gly27Asp (novel), causes “X-linked agammaglobulinemia” (MIM#300755) (3). ➢ NM_000732.4 (*CD3D*):c.128G>A, p.Trp43*, causes “immunodeficiency type 19” (MIM#615617) (3).

## Conclusions

We conclude that “BCG vaccine should be deferred until required precautions are legitimately dismissed.” In addition, the “electronic health systems” should alert vaccination providers that “There should be no contraindication to the live vaccination before a dose can be requested.” More importantly, “*parents should be well-informed and counseled that sharing critical information with the vaccination providers is essential*.” These preliminary measures are easy to implement, while awaiting wise policies on BCG vaccination (27–29).

## Data Availability Statement

The original contributions presented in the study are included in the article/supplementary material, further inquiries can be directed to the corresponding author/s.

## Ethics Statement

The studies involving human participants were reviewed and approved by Tawam Human Research Ethics Committee. Written informed consent to participate in this study was provided by the participants' legal guardian/next of kin. Written informed consent was obtained from the individual(s), and minor(s)' legal guardian/next of kin, for the publication of any potentially identifiable images or data included in this article.

## Author Contributions

A-KS, SA-H, RV, and GB: conceived, designed, and structured the report. RV: performed the variant analysis. MT: analyzed and interpreted the images. AY, AA-A, and AS: analyzed and interpreted the clinical data. A-KS, RV, and SA-H: wrote the paper. All authors contributed to the article and approved the submitted version.

## Conflict of Interest

The authors declare that the research was conducted in the absence of any commercial or financial relationships that could be construed as a potential conflict of interest.
